# A SCOPING REVIEW OF LGBTQ+ MULTIGENERATIONAL PROGRAMMING: GAPS AND OPPORTUNITIES

**DOI:** 10.1093/geroni/igad104.2377

**Published:** 2023-12-21

**Authors:** Leyi Zhou, Angela Perone

**Affiliations:** University of California, Berkeley, Berkeley, California, United States; University of California, Berkeley, Berkeley, California, United States

## Abstract

Extensive research has revealed disparities in health, housing, employment, and economics among LGBTQ+ older adults (Emlet, 2016). However, the LGBTQ+ community has a long-standing tradition of multigenerational support that can potentially alleviate some of these challenges. Innovative caregiving models and non-traditional support systems involving multigenerational networks have emerged during crises such as the AIDS epidemic of the 1980s and 1990s and the recent COVID-19 pandemic (Cahill & Valadéz, 2013; Hafford-Letchfield et al., 2022). Nevertheless, despite the growing attention to LGBTQ+ multigenerational programs, the current state of scholarly research in this area remains uncertain. To address this gap, this scoping review aims to map the literature on intergenerational interventions for the LGBTQ+ community. This study includes peer-reviewed research in English with various study designs related to different categories of intervention, program, or activity that involve LGBTQ+ participants from more than one generation. To ensure accurate and reliable results, we conducted a comprehensive search on seven databases: PsycINFO, PubMed, Web of Science, Scopus, Sociological Abstract, Embase, and CINAHL. After deleting duplicates, 130 peer-reviewed articles were identified. Preliminary findings from the scoping review reveal historical moments when research on multigenerational programming surged, which aligns with pivotal crisis moments in the LGBTQ+ community. It also underscores emerging trends in multigenerational programming, including critical inquiry about what constitutes “multigenerational” and deeper commitment to inclusion of communities of color and transgender communities. These findings provide important insights for practitioners, policymakers, and researchers to strengthen services and support for LGBTQ+ older adults.

